# A new strategy to uncover fragile X proteomic biomarkers using the nascent proteome of peripheral blood mononuclear cells (PBMCs)

**DOI:** 10.1038/s41598-021-94027-5

**Published:** 2021-07-26

**Authors:** Olivier Dionne, François Corbin

**Affiliations:** Department of Biochemistry and Functional Genomic, Faculty of Medicine and Health Sciences, Université de Sherbrooke and Centre de Recherche du CHUS, CIUSSS de l’Estrie-CHUS, Sherbrooke, QC Canada

**Keywords:** Biomarkers, Proteomics, Neurodevelopmental disorders

## Abstract

Fragile X syndrome (FXS) is the most prevalent inherited cause of intellectual disabilities and autism spectrum disorders. FXS result from the loss of expression of the FMRP protein, an RNA-binding protein that regulates the expression of key synaptic effectors. FXS is also characterized by a wide array of behavioural, cognitive and metabolic impairments. The severity and penetrance of those comorbidities are extremely variable, meaning that a considerable phenotypic heterogeneity is found among fragile X individuals. Unfortunately, clinicians currently have no tools at their disposal to assay a patient prognosis upon diagnosis. Since the absence of FMRP was repeatedly associated with an aberrant protein synthesis, we decided to study the nascent proteome in order to screen for potential proteomic biomarkers of FXS. We used a BONCAT (Biorthogonal Non-canonical Amino Acids Tagging) method coupled to label-free mass spectrometry to purify and quantify nascent proteins of peripheral blood mononuclear cells (PBMCs) from 7 fragile X male patients and 7 age-matched controls. The proteomic analysis identified several proteins which were either up or downregulated in PBMCs from FXS individuals. Eleven of those proteins were considered as potential biomarkers, of which 5 were further validated by Western blot. The gene ontology enrichment analysis highlighted molecular pathways that may contribute to FXS physiopathology. Our results suggest that the nascent proteome of PBMCs is well suited for the discovery of FXS biomarkers.

## Introduction

Fragile X syndrome (FXS) is an X-linked neurodevelopmental disorder which represents the most prevalent inherited cause of intellectual disabilities (ID) and autism spectrum disorders (ASD). Several other comorbidities are also associated with FXS, including behavioural issues such as anxiety, aggressivity and hyperactivity as well as physical and metabolic anomalies. The penetrance of those problems is highly variable, resulting in a large phenotypical heterogeneity among individuals with Fragile X (FX)^1–3^. FXS typically originate from the expansion of CGG trinucleotide repeats found in the 5’ UTR of the *FMR1* gene. The full mutation (n > 200 CGG) is associated with the epigenetic silencing of *FMR1*, and consequently, with the loss of expression of the fragile X mental retardation protein (FMRP)^4,5^.

FMRP remains the most valuable biomarker for FXS, since it can be used to identify fragile X individuals and to predict, to some extent, their cognitive functions^6–8^. Other biochemical biomarkers, including intracellular signalling pathways, polymorphisms, level of protein synthesis and the quantification of specific proteins and metabolites, have also been proposed^9,10^. From those, only a handful have been tested in clinical contexts^11–17^. Interestingly, it has been shown that the circulating level of MMP9 (Matrix Metalloproteinase 9), Ras, Hk1 (Hexokinase 1), APP (amyloid-β precursor protein) and ERK (Extracellular Regulated Kinase) phosphorylation in blood platelets can be modulated following specific treatment, thus corroborating their potential use as objective outcome measures for FXS clinical trials^11,12,14,16^. However, clinicians still have no prognostic tools at their disposal, meaning that the announcement of a FX diagnosis is accompanied by a great deal of uncertainty regarding the severity of the comorbidities presented by the affected individual. Those facts clearly illustrate one of the actual shortcomings in the management of individuals with FXS and consequently, the need for the discovery of new biomarkers to assess a patient’s prognosis upon diagnosis.

The discovery of biomarkers using shotgun proteomic approaches faces two major limitations. First, the dynamic expression range of proteins within the proteome makes identification and quantification of low abundance proteins challenging, especially in data-dependent acquisition method^18^. This fact becomes even more important when considering that weakly expressed proteins are usually critical for the characterization of physiopathological mechanisms underlying complex pathologies, such as FXS. Secondly, differentially expression analysis of large proteome dataset highlights, most of the time, a plethora of statistically significant dysregulated proteins between samples. However, not all those findings bear significance from a biological standpoint. It is therefore crucial to use an approach that promotes the identification of proteins that are both dysregulated and biologically relevant. Different workflow can be utilized to achieve such outcomes. One of the most straightforward and widely used method consists of the purification of a specific sub-proteome, either by sub-cellular compartments fractionation or by affinity chromatography. Such approaches ultimately produce proteomic samples that are both smaller, which enhance coverage of the proteome, and enriched in biologically relevant proteins^19^. Targeting the sub-proteome that best depicts the diseases-induced defects therefore constitutes the foundation of an efficient screening strategy.

FMRP is a multifunctional RNA binding protein involved in several molecular processes including regulation of histone modifications, micro-RNA interference, alternative splicing and RNA editing^20–22^. However, FMRP is mainly recognized as a translational regulator, either as a repressor or enhancer^22,23,24^. Many mRNAs subjected to FMRP translational regulation play a key role in neurodevelopment and synaptic transmission^25^. In fact, several alterations found in FXS, including the elevated number of immature dendritic spines and improper synaptic plasticity, seem related to the absence of FMRP’s translational control^26,27^. Furthermore, we recently show that the rates of protein synthesis are altered in FX peripheral blood mononuclear cells (PBMCs)^28^. These observations prompted us to use the nascent proteome, defined as proteins synthesized in a defined timeframe, for the screening of proteomic biomarkers. We hypothesize that this sub-proteome will help us to overcome problems typically encountered when performing shotgun proteomic experiments for biomarkers discovery. Indeed, we postulate that the nascent proteome, due to the translational defects encountered in FXS, will provide a proteomic signature that is well suited for such a task. Furthermore, PBMCs constitute a non-invasive model which transcriptome is known to moderately correlate with that of neuronal tissues^29^ and that can be repeatedly collected, making it ideal for our purpose.

In the present report, we used a BONCAT method coupled to label free mass spectrometry-based proteomic to purify and quantify nascent proteins produced by PBMCs. Indeed, BONCAT (Bioorthogonal Noncanonical Amino Acid Tagging) is a proteomic technique making use of the methionine surrogate l-azidohomoalanine (AHA) to enable the labelling and the purification of newly synthesize proteins^30^. It has been thoroughly used in a variety of blood cells, including PBMCs, to investigate the nascent proteome under different experimental conditions^31–34^. An adaptation of this method has also been used in the hippocampus of Fmr1 KO mice to screen for potential FXS biomarkers. Some of the candidates identified with this approach were also shown to be deregulated in the plasma of FX individuals, thus highlighting the relevance of using the nascent proteome for biomarker discovery in FXS^35^. Consequently, we used BONCAT to compare the nascent proteomes of 7 FXS males and 7 age and sex-matched controls and successfully identified differentially expressed proteins that represents potent biomarker candidates for FXS. To our knowledge, this report also constitutes the first proteomic screening for the discovery of biomarkers in native human samples of FX individuals.

## Methods

### Reagents and antibodies

Acid citric dextrose (ACD) tubes were from BD Vacutainer. Ficoll-Paque was purchased from GE Healthcare. Azidohomoalanine (AHA), bicinchonic acid assay kit, Click-it protein reaction kit, biotin alkyne probe, dithiothreitol (DTT), Triton-X100m, C18 tips, goat polyclonal anti-biotin and goat anti-rabbit Alexa FluorVR 680 IgG antibodies were from ThermoFisher. RPMI 1640 Met^-^, P8340 protease inhibitor cocktail, magnetics streptavidin beads, ammonium bicarbonate (ABC), iodoacetamide (IAA), formic acid (FA), mouse monoclonal anti-actin (clone AC-15), Donkey anti-goat IgG, and goat anti-rabbit IgG HRP-conjugated antibodies were bought from MilliporeSigma. Trypsin/lys-C and the enhanced chemiluminescence kit (ECL) were from Promega and PerkinElmer respectively. Rabbit monoclonal anti-ILK (EP1593Y), rabbit monoclonal anti-ANXA2 (ERP13052B) and mouse monoclonal anti-FERMT3 (3D6) were from Abcam. Mouse monoclonal anti-ATP2A3 (PL/IM430) was from Santa Cruz Biotechnology. Recombinant chicken anti-VCL antibody was from Immune Biosolutions. Goat anti-mouse IgG HRP-conjugated was from Jackson IR. Goat anti-mouse IRDyeVR 800CW IgG was from LI-COR Biosciences.

### Study population and ethic declarations

The study population included 7 FX patients and 7 healthy controls. Participants were all males and were matched for age. The recruitment was performed through the Fragile X Clinic, at the *CIUSSS de l’Estrie-CHUS* (Sherbrooke, Québec, Canada). Informed written consent was obtained from healthy controls and from a caregiver for FXS participants. All participants had blood draw in the morning to decrease potential diurnal variation. All experimental protocols described in this study were approved by the Ethics Review Board of the *CIUSSS de l’Estrie-CHUS* and carried out in line with the Declaration of Helsinki.

### PBMCs isolation

PBMCs isolation was carried out using Ficoll-Paque following the manufacturer instructions with some minor modifications. Briefly, blood samples were collected by venipuncture into 8 mL ACD tubes and centrifuged at 300*g* for 10 min to allow plasma collection. A volume of PBS, equal to the volume of plasma collected, was then added to each tube and resulting blood samples were placed onto a layer of Ficoll-Paque (blood/Ficoll-Paque ratio of 4:3). Afterwards, samples were centrifuged at 500*g* for 30 min. PBMCs were subsequently collected, washed two times with PBS and counted on a flow cytometer (DXH-9000 hematology analyzer, Beckman Coulter^®^).

### AHA labeling

**Figure 1:**
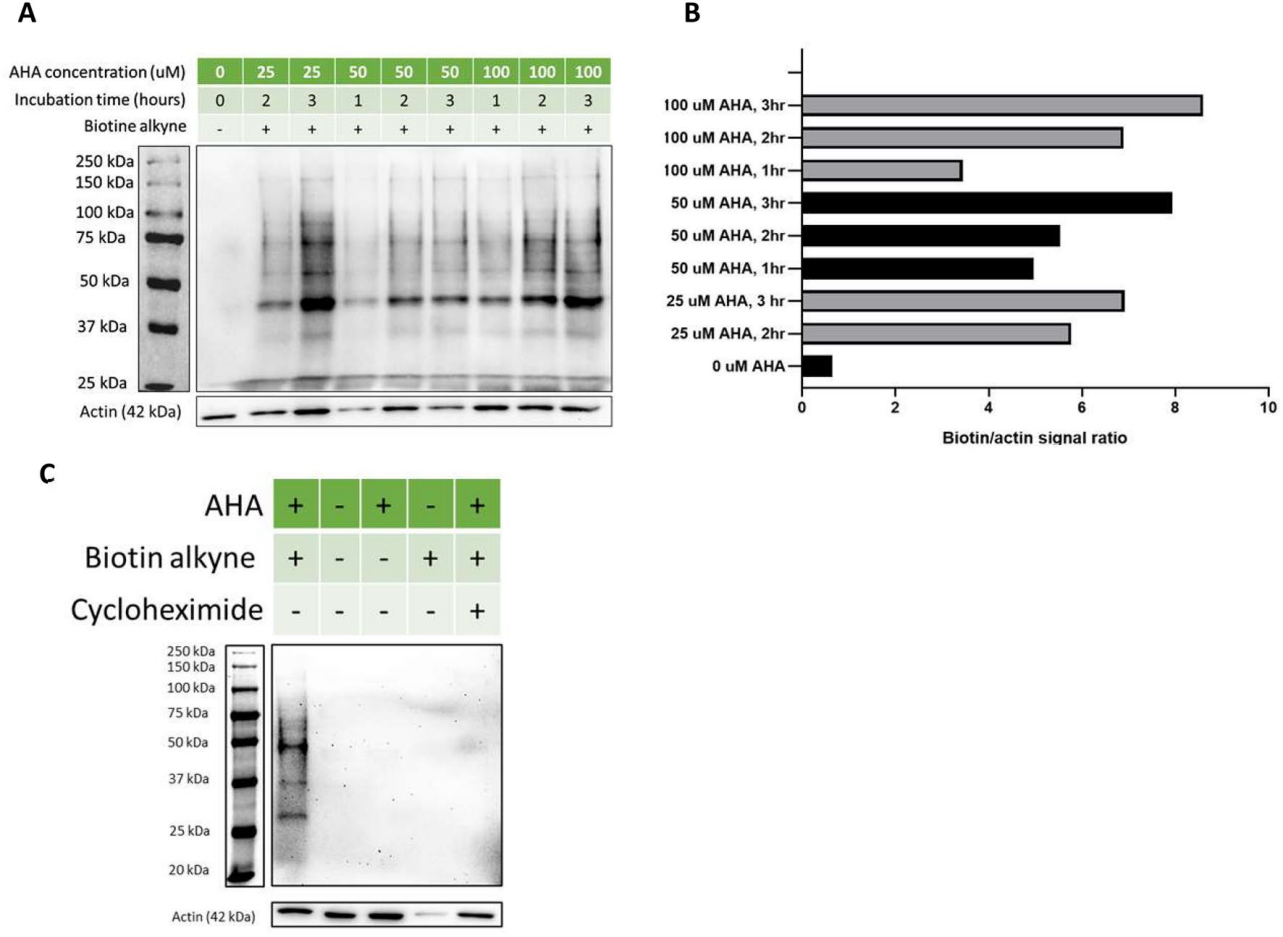
Determination of the optimal AHA labelling condition. (**A**) Anti-biotin Western Blot of protein extracts obtained from PBMCs under different AHA labelling conditions. (**B**) Quantification of the biotin signal obtained by Western blot. Biotin signal intensities were normalized with the corresponding actin signal. (**C**) Specificity of the AHA labelling and conjugation of the biotin probe towards newly synthesize proteins. Full-length blots for biotin and cropped blots for actin are displayed. Full-length actin blots are displayed in Supplementary Figure 3. Multiple exposures of blots displayed in (**A**) and (**C**) can be found in Supplementary Figures 1 and 2 respectively.

Freshly extracted PBMCs were first resuspended into warm (37 °C) RPMI 1640 Met^-^ (supplemented with 2 mM l-glutamine) and incubated for 30 min at 37 °C under gentle agitation to deplete the intracellular reserve of methionine. PBMCs were then diluted to a concentration of 3 million cells/mL and labelled with 100 μM AHA for two hours. Cells were pelleted and stored at − 80 °C after labelling. This protocol was adapted from the one previously described by another group which also perform BONCAT in PBMCs with further optimization of the AHA concentration and labelling time^32^. The optimized labelling conditions as well as the specificity of the AHA labelling and subsequent Click reaction toward nascent proteins were determined by Western blot using a specific anti-biotin antibody (Fig. 1).

### PBMCs protein extract

PBMCs were lysed in ice-cold 50 mM Tris pH 8.0, 1% NaDoc, 1% P8340 protease inhibitor cocktail and 250 U/mL Benzonase nuclease. After 15 min on ice, cells were homogenized with a 28G needle and proteins were pelleted for 25 min at 20,000*g* (4 °C). Protein quantification in the supernatant was determined by bicinchonic acid assay.

### Isolation of nascent proteins and preparation for MS analysis

Two experimental groups were formed (control and FX) by combining an equal amount of protein from each participant. The conjugation of the biotin probe was performed on 150 μg of pooled proteins using the Click-it protein reaction kit, following the manufacturer instructions and using a biotin alkyne probe. Nascent biotinylated proteins were purified by an overnight incubation with magnetics streptavidin beads at 4 °C. The beads were washed 4 times with PBS containing 0.1% Triton X-100 and 5 times with a 20 mM pH 8.0 ABC (ammonium bicarbonate) buffer. Cysteines reduction was realized by incubating the beads with 10 mM DTT at 60 °C for 30 min and subsequent alkylation with 15 mM iodoacetamide for 30 min at room temperature in the dark. Proteins digestion into peptide was performed overnight at 37 °C with 1 μg of trypsin/lys-C. The digestion was stopped by adding formic acid to a final concentration of 1%. The supernatant was transferred to a clean tube and the beads were washed twice with 60% acetonitrile in 0.1% formic acid. All supernatants were combined and evaporated. The peptide samples were reconstituted in 0.1% trifluoroacetic acid, desalted with a C18 tip and dried. Nascent proteins were suspended in 1% formic acid and kept at − 20 °C. Four technical replicates were made for each experimental group, and each replicate was injected twice into the mass spectrometer (intra-assay replicate).

### Preparation of PBMCs total proteome for MS analysis

The same experimental groups used in the previous section were also used for the preparation of the total proteome. Briefly, 5 μg of pooled protein extracts were solubilized with 8 M urea in 10 mM HEPES pH 8.0 (protein/urea ratio (p/v) of 1:1). Reduction of proteins was performed with 10 mM DTT at 60 °C for 30 min and alkylation with 15 mM IAA at room temperature in the dark for 30 min. Urea concentration was lowered below 1 M by adding 50 mM pH 8.0 ABC buffer. Proteins digestion into peptide was performed overnight at 37 °C with 0.25 μg (ratio trypsin/protein of 1:20) of trypsin/lys-C and stopped by adding 1% formic acid. Peptide samples were evaporated, desalted with a C18 tip and evaporated. The total proteome was suspended in 1% formic acid and kept at − 20 °C. Four technical replicates were made for each experimental group, and each replicate was injected twice into the mass spectrometer (intra-assay replicate).

### LC–MS/MS analysis

Peptides were injected into an HPLC (nanoElute, Bruker Daltonics) and loaded onto a trap column with a constant flow of 4 µL/min (Acclaim PepMap100 C18 column, 0.3 mm id × 5 mm, Dionex Corporation) and eluted onto an analytical C18 Column (1.9 µm beads size, 75 µm × 25 cm, PepSep). Peptides were eluted over a 2-h gradient of acetonitrile (5–37%) in 0.1% formic acid at 400 nL/min while being injected into a TimsTOF Pro Mass Spectrometer equipped with a Captive Spray nano-electrospray source (Bruker Daltonics). Data were acquired using data-dependent auto-MS/MS with a 100–1700 m/z mass range, with PASEF enabled with a number of PASEF scans set at 10 (1.27 s duty cycle) and a dynamic exclusion of 0.4 min, m/z dependent isolation window and collision energy of 42.0 eV. The target intensity was set to 20,000, with an intensity threshold of 2500.

### Mass spectrometry data analysis

Proteins identification from the raw data was accomplished with the MaxQuant software^36^ (version 1.6.10.0). Peaks list was searched against the Uniprot human database (09/2019)^37^. Trypsin was set as digestion enzyme with specificity for arginine and lysine (but not before proline). A maximum of two miss cleavages was tolerated. Oxidation of methionine and acetylation of proteins N-terminus were set as variable modifications, while carbamidomethylation of cysteine was set as a fixed modification. Carbamylation of lysine was set as variable modification only for the analysis of the total proteome. The false discovery rate of peptides (minimum of 7 amino acids) and proteins were set to 0.05 using a reverse database. The mass tolerance was set to 7 ppm for precursor ions and 20 ppm for fragment ions. The MaxQuant label-free quantification (LFQ) was used, with a minimum ratio count of two, for accurate intensity based quantification of proteins between samples^38^.

### Proteomic data processing

Processing of proteomics data was carried out with the Perseus software^39^ (version 1.6.7.0.). Proteins identified as "potential contaminants", "only identified by site" or "reverse" by MaxQuant were excluded. Data were normalized (average intensities of each sample equal within each group) before further analyzes. Proteins only identified in at least 50% of the samples from each group with a minimum of 1 unique peptide and 2 total peptides were kept. The statistical significance of protein expression between the two groups was evaluated by a two-tailed Student *t* test, carried out in the Perseus software (p < 0.05 for the nascent proteome; p < 0.01 after correction with a permutation-based FDR, for the total proteome analysis). We choose to be more stringent for the differential expression analysis carried out for the total proteome to lower the numbers of statistical hits. The corresponding volcano plots were drawn using the ggplot2 package in R^40,41^.

For the nascent proteome analysis, a "negative control" was produced to eliminate contaminating and endogenous biotinylated proteins. To achieve this, an unlabelled protein extract from a control PBMCs was subjected to all the steps of the workflow describe above (Click reaction, streptavidin pull down, on beads proteins preparation for MS analysis, etc.). Proteins identified with a fold change (experimental sample/negative control) superior to 1.1 were kept for further analysis. Proteins that do not fulfill this criterion where excluded.

### Bioinformatic analysis

Functional annotation enrichment analyses were carried out with the Panther classification system (http://www.pantherdb.org) using the Gene Ontology, Panther protein class and REACTOME pathway annotations sets^42–44^. Protein–protein interaction networks (PPI) were obtained from the web-based LENS tool (Lens for Enrichment and Network Studies of Proteins) at the website: http://severus.dbmi.pitt.edu/LENS^45^.

### Western blots

Optimal AHA labelling conditions and specificity towards newly synthesized proteins were confirmed by an anti-biotin Western blot (Fig. 1). Briefly, 10 μg of protein samples were resolved on a 10% SDS-PAGE, transferred onto a nitrocellulose membrane, block with 5% non-fat dry milk and incubated with the following antibodies: anti-biotin (1:1000) and anti-actin (1:5000). Blots were revelated using an enhanced chemiluminescence (ECL) kit and imaged with ChemiDoc (Bio-Rad).

Western blots were also used to validate 5 proteins found deregulated in fragile X PBMCs by the proteomic screening (ILK, ATP2A3, ANXA2, FERMT3 and VCL). Briefly, 15 μg of proteins from each participant were resolved on a 9% (ILK, ATP2A3 and VCL) or a 12% (ANXA2 and FERMT3) SDS-PAGE, transferred onto a nitrocellulose membrane, block with 5% non-fat dry milk and incubated with the following antibodies: anti-ILK (1:2000), anti-ANXA2 (1:2000), anti-ATP2A3 (1:250), anti-FERMT3 (1:2000), anti-VCL (1:1000) and anti-actin (1:5000).Anti-goat IgG (1:10,000), anti-mouse IgG (1:10,000) and anti-rabbit IgG (1:10,000) HRP-conjugated secondary antibodies were used for ECL revelation, while anti-mouse IRDyeVR 800CW IgG (1:10,000) and anti-rabbit Alexa FluorVR 680 IgG (1:10,000) were used for fluorescence-based immunostaining. All immunoblots were analyzed with the Image-J software (NIH)^46^. Immunoblots were revelated either by ECL (imaged with ChemiDoc, BioRad) or by fluorescence (imaged with the Odyssey Infrared Imaging System LI-COR Biosciences).

### Statistical analyses

Fisher exact tests were used with R studio to perform Gene Ontology enrichment analysis. A p-value inferior to 0.05 (corrected with a permutation-based FDR) was considered significative. For the Western blot analysis, statistically significant difference was determined by a two-tailed Student *t* test calculated in GraphPad Prism (version 8.3.0 for Windows, GraphPad Software, San Diego, California USA, http://www.graphpad.com). A p-value inferior to 0.05 was considered significative.

## Results

### Population characteristics

The study population included 7 fully mutated males with fragile X (mean age of 28.7 ± 9.0) and 7 healthy males (mean age of 27.7 ± 8.1). Individual characteristics of each participant, including age, medication and fragile X diagnostic are listed in Table 1.

**Table 1:** Individual characteristics of each participant.

ID	Age	Sex	Medication	Fragile X diagnostic
**Fragile X cohort**				
X1	25	Male		Full mutation
X2	40	Male	Levothyroxine, antipsychotic (Olanzapine)	Full mutation
X3	17	Male		Full mutation
X4	29	Male		Full mutation
X5	43	Male		Full mutation
X6	30	Male	Anti-diabetic (Metformin)	Full mutation
X7	25	Male		Full mutation
**Control cohort**				
C1	24	Male		NA
C2	23	Male		NA
C3	41	Male	Antiandrogen (Finasteride)	NA
C4	26	Male		NA
C5	18	Male		NA
C6	34	Male	Levothyroxine	NA
C7	35	Male		NA

### The nascent proteins identified are enriched in functional annotations associated with RNA metabolism

**Figure 2:**
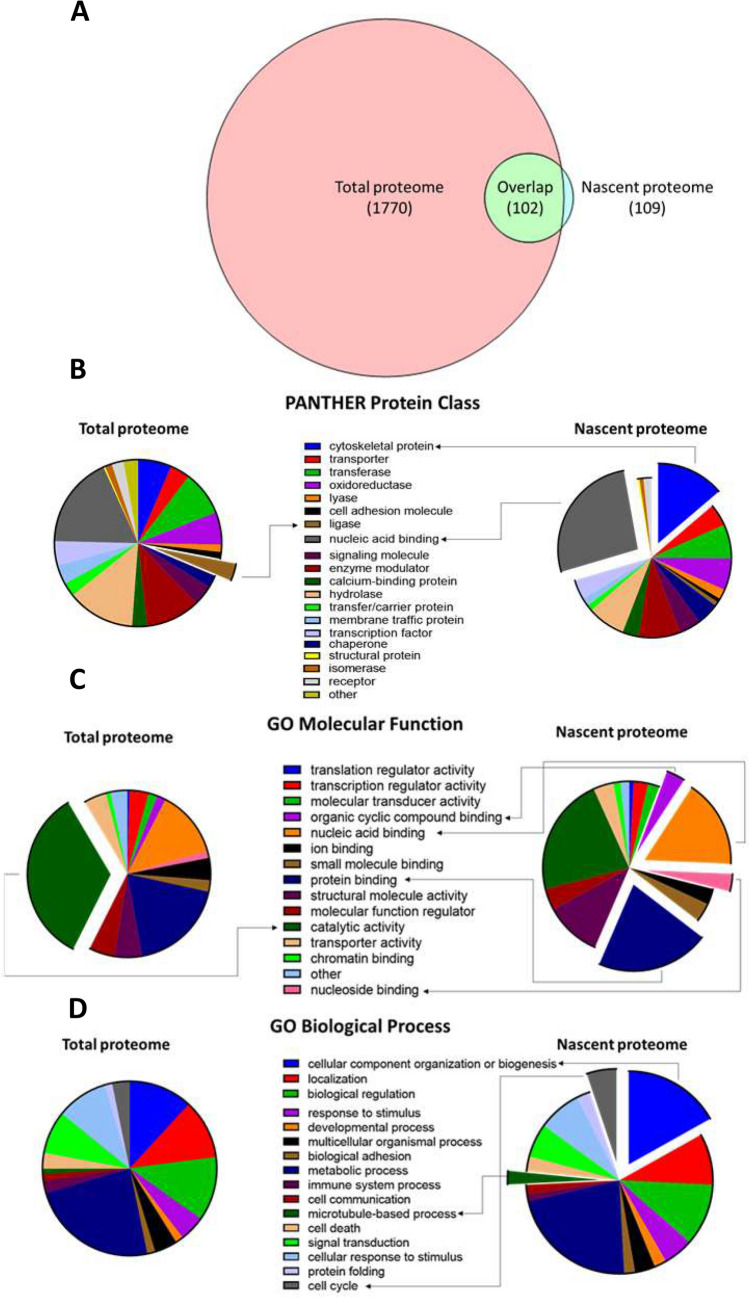
Proteins identified in the nascent and total proteome show distinct features. Far less nascent proteins were identified. (**A**) Venn diagrams representing the total number of proteins identified (FX and CTL combined) in the proteomic screening. When compared to the proteins identified in the total proteome, nascent proteins identified in this study presents an enrichment for annotations with specific Gene Ontology terms. A fisher exact test was used to determine enriched annotations between the two proteomes. FDR inferior to 0.05 was considered significative. Pie charts representing the distribution of different annotations between the two proteomes: PANTHER protein class (**B**), Gene Ontology molecular function (**C**) and biological process (**D**). The exposed part represents enriched annotations in the corresponding proteome. The Venn diagram was made using the Venn Diagram Plotter software (version 2.0, https://omics.pnl.gov/software/venn-diagram-plotter).

As shown in Fig. 2A, a smaller number of nascent proteins were identified. Moreover, 7 of the 109 newly synthesized proteins identified were not found among the 1770 proteins within the total proteome. Intriguingly, 5 of those 7 proteins are found deregulated in fragile X PBMCs (Table 2). Moreover, these nascent proteins display an enrichment for functional annotations related to nucleic acid, nucleosides, and protein binding as well as in cellular component organization or biogenesis (Fig. 2B–D). Several of these annotations can be associated with RNA metabolism, suggesting that the nascent proteins identified in this study may be involved in the same molecular processes as FMRP. Taken together, these results support our hypothesis that the isolation of nascent proteins will promote the identification of dysregulated proteins between FX patients and control individuals that are also relevant from a biological standpoint.

**Table 2:** Differentially expressed proteins in the fragile X nascent proteome. Thirty proteins are found differentially expressed (p < 0.05) in the nascent proteome of the fragile X group. From those proteins, 11 are also found deregulated in the total proteome (q < 0.01) of the fragile X group and 5 where only detected in the nascent proteome.

Gene names	Protein names	Fold change nascent proteome	p-value nascent proteome	Fold change total proteome	q-value total proteome
AHNAK	Neuroblast differentiation-associated protein AHNAK	0.087	< 0.0001	0.704	< 0.0001
ATP2A3	Sarcoplasmic/endoplasmic reticulum calcium ATPase 3	0.193	0.048	1.168	< 0.0001
PDIA3	Protein disulfide-isomerase A3	0.288	0.003	1.121	< 0.0001
PF4	Platelet factor 4	Detected only in the control group	0.037	1.252	< 0.0001
TLN1	Talin-1	1.497	0.024	1.369	< 0.0001
FERMT3	Fermitin family homolog 3	1.412	0.001	1.202	< 0.0001
HIST1H4A	Histone H4	2.861	< 0.0001	1.188	< 0.0001
ILK	Integrin-linked protein kinase	1.684	0.034	1.342	< 0.0001
MPO	Myeloperoxidase	5.157	0.001	1.115	< 0.0001
ANXA2	Annexin A2	2.735	0.037	0.818	< 0.0001
VCL	Vinculin	3.013	0.034	1.330	< 0.0001
SPP2	Secreted phosphoprotein 24	0.131	0.045	Not detected	NA
HIST1H2AC; HIST3H2A	Histone H2A type 1-C; Histone H2A type 3; Histone H2A type 1-B/E	2.361	< 0.0001	Not detected	NA
PRPF40A	Pre-mRNA-processing factor 40 homolog A	3.052	< 0.0001	Not detected	NA
PRPF38B	Pre-mRNA-splicing factor 38B	19.741	< 0.0001	Not detected	NA
AP3D1	AP-3 complex subunit delta-1	Detected only in the fragile X group	0.028	Not detected	NA
GNAI2	Guanine nucleotide-binding protein G(i) subunit alpha-2	0.487	0.016	0.993	ns
NCL	Nucleolin	0.402	0.035	1.045	ns
DEK	Protein DEK	0.414	0.015	0.853	ns
H1FX	Histone H1x	0.436	0.017	1.186	ns
RAB1B; RAB1C	Ras-related protein Rab-1B; Putative Ras-related protein Rab-1C	Detected only in the control group	0.007	1.161	ns
SNRPD3	Small nuclear ribonucleoprotein Sm D3	Detected only in the control group	0.014	1.009	ns
SNRPE	Small nuclear ribonucleoprotein E	0.147	0.008	0.833	ns
RPL30	60S ribosomal protein L30	Detected only in the control group	0.035	0.828	ns
RPL7	60S ribosomal protein L7	2.799	0.022	0.972	ns
H2AFY	Core histone macro-H2A.1	2.257	0.010	1.007	ns
NOP58	Nucleolar protein 58	2.680	0.015	1.049	ns
RPS18	40S ribosomal protein S18	3.767	0.024	0.955	ns
PCBP1	Poly(rC)-binding protein 1	Detected only in the fragile X group	0.046	1.330	ns
TOP1	DNA topoisomerase 1	Detected only in the fragile X group	0.019	0.547	ns

### Differentially expressed proteins are found in PBMCs nascent and total proteome

**Figure 3:**
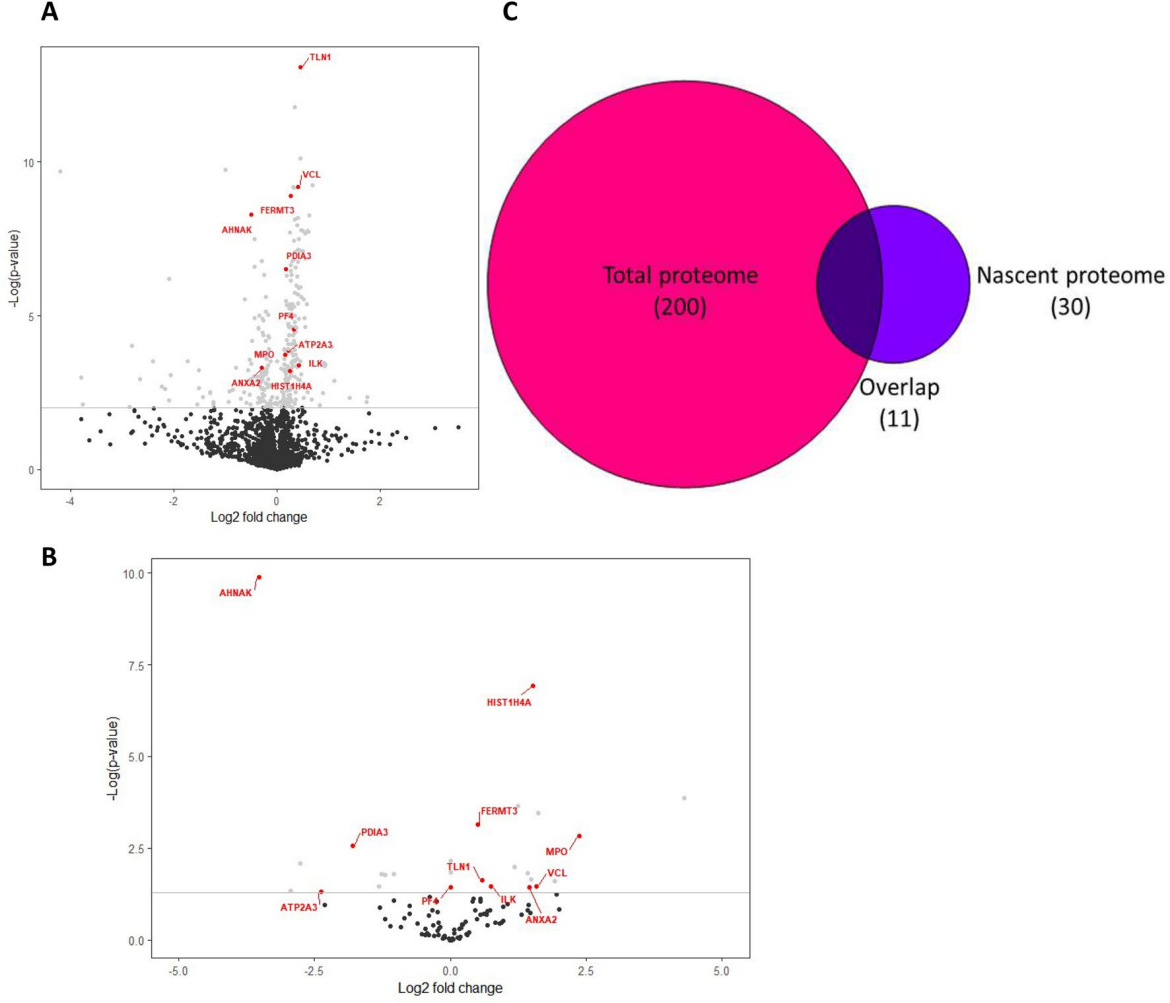
Differentially expressed proteins are found in fragile X PBMCs. (**A**) Volcano plot of the proteins identified in the total proteome of PBMCs. The horizontal bar represents a p-value of 0.01, which was considered significative. (**B**) Volcano plot of the proteins identified in the nascent proteome of PBMCs. The horizontal bar represents a p-value of 0.05, which was considered significative. The proteins marked in red are found to be differentially expressed in both proteomes. (**C**) Venn diagram of the number of differentially expressed proteins found in both fragile X nascent and total proteome. This graph was made using the Venn Diagram Plotter software (version 2.0, https://omics.pnl.gov/software/venn-diagram-plotter).

Analysis of the total proteome successfully identified 1770 distinct proteins. More precisely, 1752 and 1768 were identified in fragile X and control individuals respectively (Supplementary Table S1). Of these proteins, 200 were found to be deregulated in FXS samples (Fig. 3A). Indeed, 135 were found to be upregulated while 65 were found downregulated (Supplementary Table S2). On the other hand, the BONCAT approach made it possible to identify a total of 109 nascent proteins, 105 from the FXS patients and 106 from control samples (Supplementary Table S3). The total number of nascent proteins identified was comparable to that of a previous study which also used BONCAT in PBMCs with similar labelling conditions^32^. Thirty nascent proteins were found deregulated in FX PBMCS (Fig. 3B), of which 17 were upregulated and 13 downregulated (Table 2).

Moreover, 11 dysregulated proteins were identified using both approaches. The trend of the expression of 7 of those 11 proteins was shown to be constant between the nascent and total proteomes. More specifically, TLN1, FERMT3, HIST1H4A, ILK, MPO and VCL were constantly upregulated, while AHNAK was downregulated. However, ATP2A3, PDIA3, PF4 and ANXA2 presented opposite trend of perturbation between the nascent and the total proteome (Fig. 3C and Table 2).

### Bioinformatic analysis of the differentially expressed proteins

**Figure 4:**
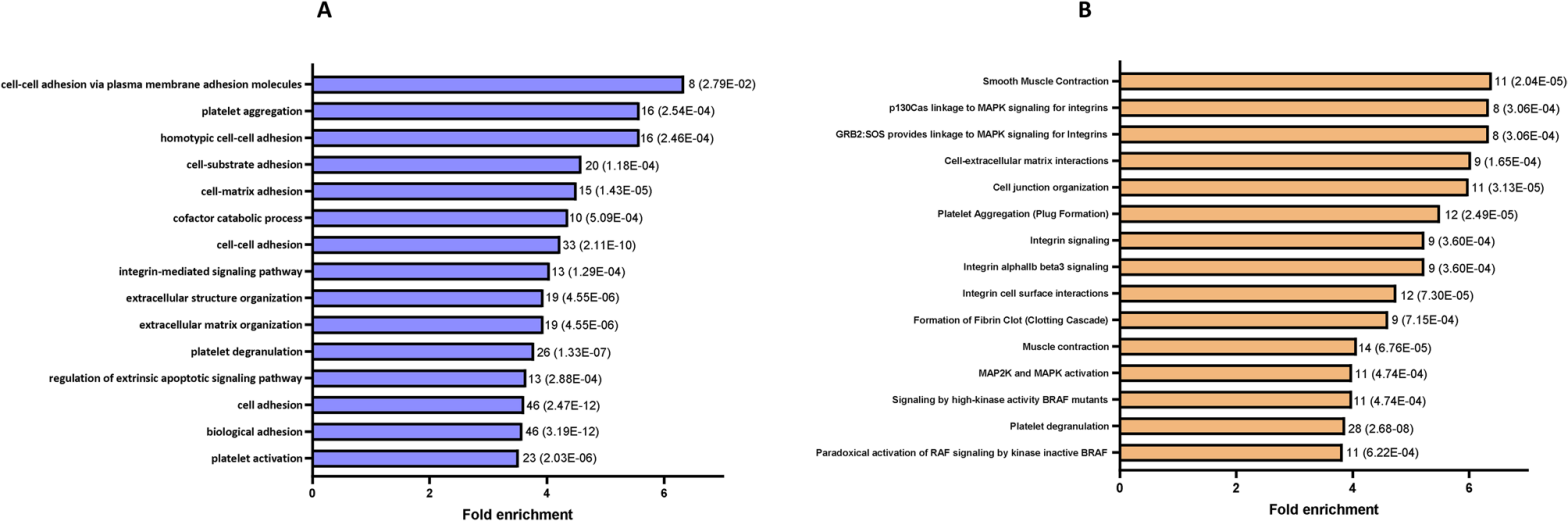
Functional annotation enrichment analysis of the differentially expressed proteins found in fragile X PBMCs total proteome. The number of proteins associated and corresponding FDR for each annotation are represented beside the bar. (**A**) The top 15 gene ontology biological process annotations ranked by the fold enrichment. (**B**) The top 15 REACTOME annotations ranked by the fold enrichment.

Functional annotation enrichment analysis of the differentially expressed proteins found in the total proteome of FX PBMCs was carried out with PANTHER. We used all the 1770 proteins identified in the total proteome as reference. Results showed that dysregulated proteins are involved in cellular adhesion, platelet aggregation and degranulation, MAPK2 and MAPK activation and in signalling pathways related to the MAPK and integrins (Fig. 4).

**Figure 5:**
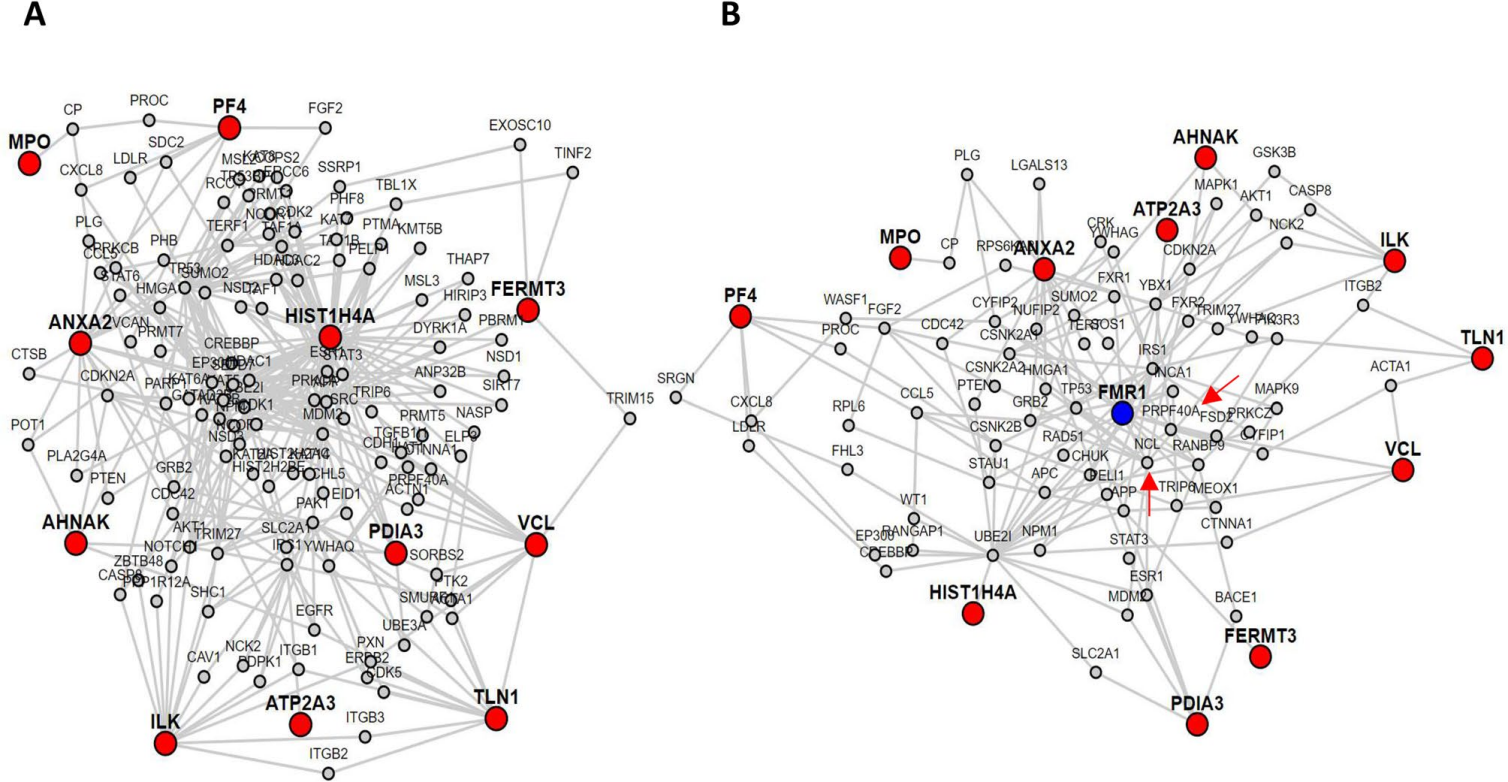
Protein–protein interaction networks of 11 proteins found differentially expressed in both nascent and total proteome of fragile X PBMCs. (**A**) Interaction network corresponding only of the 11 candidates (red). (**B**) Interaction network generated with the 11 candidate proteins (red) and FMRP (blue) set as target. The NCL and PRPF40A proteins, which are found deregulated in FXS nascent proteome, are also present in this network (red arrows).

A PPI network of the 11 proteins found differentially expressed in FX nascent and total proteome (Table 2) is illustrated in Fig. 5A. This network shows that these proteins have common interaction partners, strongly suggesting a potential interaction between them and their involvement in the same biological pathways. A second PPI network was generated using the same 11 proteins set as candidates and with the FMR1 gene set as target. As illustrated in Fig. 5B, all proteins were found to be enriched in this network, showing that the 11 candidates possess common interaction partners with FMRP. This result suggests the implication of those 11 proteins in molecular mechanism in which FMRP is involved, and consequently, the involvement of their deregulation in the mechanism underlaying FXS physiopathology. It is also noteworthy that two proteins (NCL and PRPF40A) differentially expressed in the nascent proteome are present in the interaction network containing FMRP and the 11 candidate proteins.

All those observations are supported by the statistics associated with the LENS analysis (Supplementary Table S4), in which three values are used to describe the network connectivity. These values include "minimum shortest path length", "average shortest path length" and the number of "disconnected nodes". Those results support the fact that the two networks are well enriched. Indeed, all three values are less important in "candidates" when only the 11 candidates are given has well as in "candidate to target" when compared to the values associated with "candidates to random" and "target to random".

### Validation of candidate proteins by western blot

**Figure 6:**
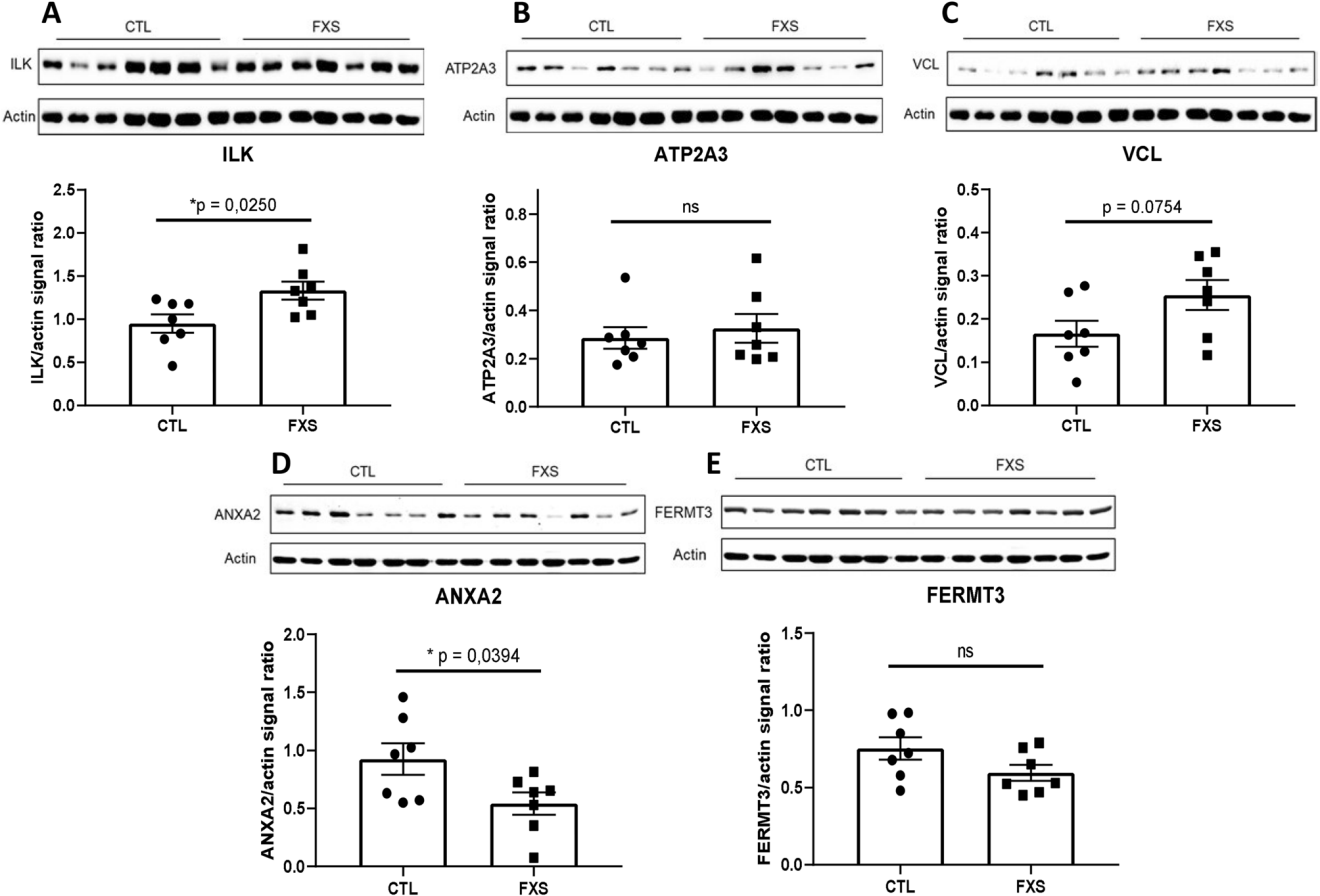
Validation of 5 candidate proteins by western blot. (**A**) Integrin-linked protein kinase (ILK). (**B**) Sarcoplasmic/endoplasmic reticulum calcium ATPase 3 (ATP2A3). (**C**) Vinculin (VCL). (**D**) Annexin A2 (ANXA2). (**E**) Fermitin family homolog 3 (FERMT3). (**F**) Signals of all proteins were normalized to the actin signal to account for loading disparity. A two-tailed Student *t* test was used to calculate the significance of the difference measured between the two groups. Cropped blots are displayed. For full-length blots, refer to Supplementary Figure 4.

Five of the 11 proteins differentially expressed in both nascent and total proteome (ILK, ATP2A3, ANXA2, FERMT3 and VCL) from pooled samples of FX PBMCs were chosen to be validated by Western blot (Fig. 6). The trend of perturbation of ILK, ATP2A3, ANXA2 and VCL measured by Western blot was consistent with that measured by mass spectrometry. Furthermore, the difference measured for ILK (p = 0.0250) and ANXA2 (0.0394) were found to be statistically significant. Full-length blots are available in Supplementary Figure 4.

## Discussion

The discovery of biomarkers using shotgun proteomic workflow recurrently led to the identification of a high number of protein candidates. Most of them might ultimately be irrelevant, meaning that a lot of time and resources are spent on selection and validation of newly identified candidates before using them in a clinical setting. This is particularly true when screening experiments are conducted on human biological samples. Indeed, disease-induced heterogeneity and interindividual variability inherent to the use of such samples both add an extra level of intricacy to this already complex equation. One way to limit these drawbacks involves the elaboration of a comprehensive targeted strategy and a rigorous experimental procedure. Indeed, a careful selection of affected individuals and the use of a subproteome that specifically aims at the biological defects induced by the studied pathology can lead to the identification of a higher yield of valuable biomarkers.

In the current study, we deliberately selected 7 fully mutated FXS males in order to restrain the etiologic heterogeneity within our FX cohort. The inclusion of mosaic or female patients (which both expressed variable level of FMRP) would have greatly increased the variability of our proteomic analysis^6,7^. Furthermore, we chose to study the nascent proteome to screen for proteomic biomarkers, since it is well established that the absence of FMRP leads an aberrant protein synthesis. Moreover, as shown by the functional annotation enrichment analysis (Fig. 2C,D), the proteins identified in the nascent proteome might be well adapted to portray the consequences of FMRP’s absence on the various molecular processes in which it is involved, especially those associated with RNA (regulation of translation, alternative splicing, micro-RNA interference and RNA editing)^20,21,22^. Indeed, newly synthesis proteins identified in this study present enrichment for annotations that can be related to RNA metabolism, such as: nucleic acid binding, nucleoside binding and organic cyclic compounds binding^39,40^. Our strategy successfully highlighted 30 dysregulated proteins among the 109 nascent proteins identified from FX individuals PBMCs. This subset approximately represents 28% of all nascent proteins identified in this study (Table 2). Moreover, the mRNA of 20 of those 30 proteins were shown to be bound by FMRP^47,48^. Considered as a whole, these results withstand PBMC’s nascent proteome as a promising way to identify potential biomarkers for FXS.

It is well known in the proteomic field that different strategy leads inevitably to the identification of different sets of candidates. Remarkably, more than 35% of proteins found deregulated in the nascent proteome of FX were also shown to be deregulated in our total proteome analysis elevating these 11 proteins as biomarker candidates (Fig. 2A and Table 2). Interestingly, 4 of these 11 proteins showed the opposite trend of perturbation between the nascent and total proteomes. These differences could arise from an alteration of their turnover rates, which in turn may result from FMRP’s absence. As such, the perturbation observed in the nascent proteome can be indicative of the level of synthesis of a specific protein, while the trend observed in the total proteome may reflect the level of catabolism. In this sense, ANXA2 (which was upregulated in the nascent proteome but downregulated in the total proteome), may present both higher levels of synthesis and degradation in PBMCs of FX individuals. An altered protein turnover rate may also explain the substantial difference regarding the fold changes of the 30 dysregulated proteins in the nascent proteome of FX PBMCs. Moreover, these opposite trends of perturbation between the two proteomes could also be the result of a compensatory mechanism, in which the level of synthesis of a protein is adjusted to compensate for its abnormal expression in the total proteome.

We further validated the dysregulated expression of 5 of those 11 candidates (ILK, ATP2A3, ANXA2, FERMT3 and VCL) by Western blot using the total proteome of PBMCs. Furthermore, as supported by the PPI network (Fig. 5B), many of those 11 proteins can be associated, because of their function and interaction, to the biological process involved in FXS physiopathology.

One of those validated candidates is the ANXA2 protein. The latter inhibits the degradation of LDL receptors meditated by the Proprotein convertase subtilisin/kexin type 9 (PCSK9). Such function is achievable through the binding of ANXA2 to PCSK9, which can occur in both an intra and extracellular manners^49–51^. We have previously found a high rate of hypocholesterolaemia in the FX population^3^. Furthermore, there was no correlation between the plasma level of PCSK9 and LDL cholesterol in FX individuals, a phenomenon which is, however, observed in healthy controls^3,52^. Here we report a dysregulation of ANXA2 expression in FXS which provides new insights of the underlying mechanism of hypocholesterolaemia reported in this population. Further studies are warranted to validate the interaction between FMRP, ANXA2, PCSK9 and cholesterol levels. ANXA2 is also known to interact with S100A10 and AHNAK (another candidate of this study) to form a complex that increase cell surface expression of L-type Voltage-gated calcium channels in mouse brain^53^. AHNAK also promotes the activity of the Raf/MEK/ERK signalling cascade, a pathway hyperactivated in the brain of KO mice and in FX patients blood cells^11,54–57^. Interestingly, a recent study has found deregulation in the expression of AHNAK in PBMCs of children with idiopathic ASD, suggesting the deregulation of AHNAK as a shared pathophysiological mechanism of both conditions^58^.

Three others validated candidates (FERMT3, ILK and VCL), along with TLN1 (another candidate of this study), are involved in integrin activation and/or subsequent signal transduction^59–61^. Furthermore, the functional annotation enrichment analysis (Fig. 4) shown that the proteins deregulated in FX PBMCs are associated with biological processes related to cell adhesion and, consequently, in integrins signalization and underlaying signalling cascades. Taken together, those observations suggest an alteration of integrin mediated signalling in FXS. Integrins activation leads to a multitude of cellular processes which are transduced by many signalling pathways, such as the Raf/MEK/ERK and PI3K/AKT/mTOR cascades^61–66^, both of which are known to be upregulated in KO mice and in cells derived from FX individuals^11,49–52,62–64,67^. Integrins are also involved in many neuronal processes, including neurite outgrowth, synapse formation and synaptic transmission^70–72^. Impairing integrin binding to the extracellular matrix (ECM) with RGD peptides (a sequence recognize by integrin extracellular domains) reduced synaptic strength by promoting a decrease in post-synaptic AMPA receptor expression^65,66,68–70^. In neuronal tissue, RGD peptides can be released by the enzymatic activity of the metalloproteinase 9 (MMP9) towards the components of the ECM^71–75^. The overexpression of MMP9 found in the brain of Fmr1 KO mice and in FXS patients^12,71,76–78^ can therefore be associated with the impaired synaptic plasticity observed in animal models of the disorder^79–82^. These observations, along with dysregulation of FERMT3, ILK, TLN1 and VCL in fragile X PBMCs suggest that deregulation of integrin activity plays a role in FXS molecular physiopathology and that peripheral blood cells, such as PBMCs or platelets, are relevant models to address this hypothesis^54,79,83–85^.

Characterization of the nascent proteome has been previously carried out in the mouse model of FXS. Indeed, Bowling and al utilized an adaptation of the BONCAT technique to identify newly synthesis proteins in Fmr1 KO and WT mice hippocampus under basal and stimulated conditions. They identified several candidates, of which 3 were also shown to be deregulated in the plasma of FXS individuals^35^. A subsequent study validated 2 of those previously identified candidates (along with MMP9) during a clinical trial, showing that their modulation is correlated with treatment efficacy, thus establishing the relevance of the nascent proteome of mice brain to screen for FXS biomarkers^16^. Our present report supports the use of the nascent proteome for biomarker discovery using ex vivo native cells from FX individuals. The use of a human model makes it possible to take into consideration the polymorphisms specific to each patient and consequently reflect the phenotypic heterogeneity found within the FXS population. Such benefit cannot be achieved with animal models of FXS, since KO animals share a highly similar genetic background between each other^86^. Furthermore, PBMCs can be repeatedly collected and be used to monitor the effect of disease-modifying drugs on the nascent proteome during clinical trials. However, it should be noted that despite the employment of a similar experimental strategy, only one differentially expressed nascent protein (ANXA2) identified in this study was also dysregulated in the nascent proteome of KO mice hippocampus^35^.

The principal limitation of our study consists of the somewhat low number of proteins identified, especially in the nascent proteome. One possible explanation for this drawback resides in the variations regarding turnover rates between different proteins. Some of the most abundant proteins (ex: histones, cytoskeletal, heat shock proteins, etc.) presents very high turnover rate^87^, which means that they can hinder the identification of low abundant proteins in the nascent proteome. The two hours timeframe used to label nascent proteins may also have contributed to reducing the number of proteins identified since it favours only proteins with high turnover rate. The concentration of AHA used in this study may also hamper our ability to identify nascent proteins. Indeed, previous studies reported deeper coverage of the nascent proteome by using higher concentration of AHA^33,88^. Despite these potential limitations, the number of nascent proteins identified in PBMCs is similar to the one reported by another study using similar experimental conditions^32^. Our experimental workflow, which consisted of the processing of pooled samples, can also have limited the scope of our analysis by limiting our capacity to individually assess each participant’s unique proteome. The quantitative aspect of this study is also limited, as it is the case for all label-free based shotgun proteomic experiments. As such, all the candidates identified in this study will have to be extensively validated in a larger population before being confirmed as FX biomarkers.

## Conclusion

We took advantage of the known translational defects caused by the absence of FMRP to identify several potential biomarkers in FXS. Furthermore, our strategy allows for a minimal recruitment of patients, which limited the inter-individual variation within our FX cohorts, a known caveat of human samples. Obviously, further validation of those candidates in a larger FX cohort in relation to clinical profile is warranted. Nevertheless, the strategy put forth in the present study clearly indicates the feasibility, even for rarer disease, to uncover biomarkers using pathology-driven sub-proteomic strategies with limited human samples.

## Supplementary Information

Supplementary Figures.

Supplementary Tables.

## Data Availability

All data generated or analyzed during this study are included in this article. If any additional information is required, it may be obtained by request from the corresponding author.
