# Supplementary material for: A new strategy to uncover fragile X proteomic biomarkers using the nascent proteome of peripheral blood mononuclear cells (PBMCs)
Source: Sci Rep. 2021 Jul 26;11:15148. doi: 10.1038/s41598-021-94027-5 (PMC8313568; doi:10.1038/s41598-021-94027-5)
Supplement: Supplementary file 1 — Supplementary Figures. [file 41598_2021_94027_MOESM1_ESM.pdf]

A New Strategy to Uncover Fragile X Proteomic Biomarkers Using the Nascent Proteome of Peripheral Blood Mononuclear Cells (PBMCs)

Supplementary Figures

Olivier Dionne[1*] and François Corbin[1*]

[1]Department of Biochemistry and Functional Genomic, Faculty of Medicine and Health Sciences, Université de Sherbrooke and Centre de Recherche du CHUS, CIUSSS de l'Estrie-CHUS, Sherbrooke, Quebec, Canada

[*]**Correspondence:**

Olivier Dionne: olivier.dionne@usherbrooke.ca ;

François Corbin: francois.corbin@usherbrooke.ca

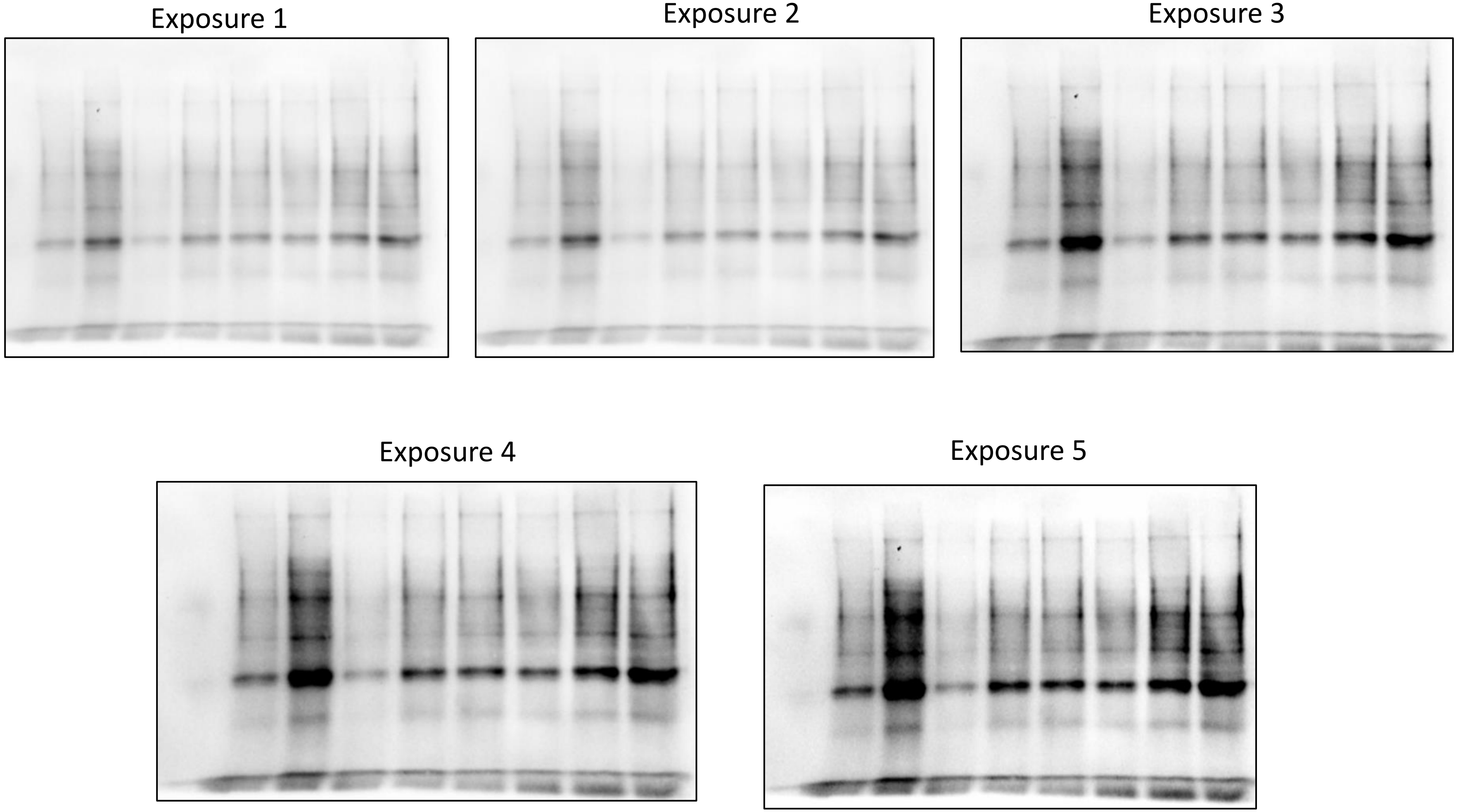

Supplementary Figure 1 : Multiple exposures of the anti-biotin western blot displayed in Figure 1A. Exposure 3 is displayed in Figure 1A.

Anti-biotin western blot

Exposure 1 | Exposure 2 | Exposure 3

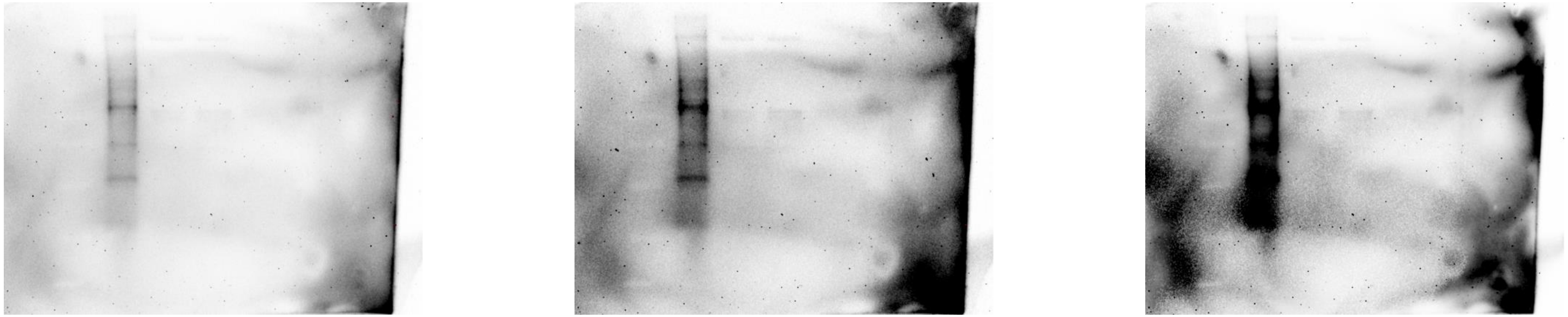

Supplementary Figure 2 : Multiple exposures of the anti-biotin western blot displayed in Figure 1C. Exposure 2 is displayed in figure 1C.

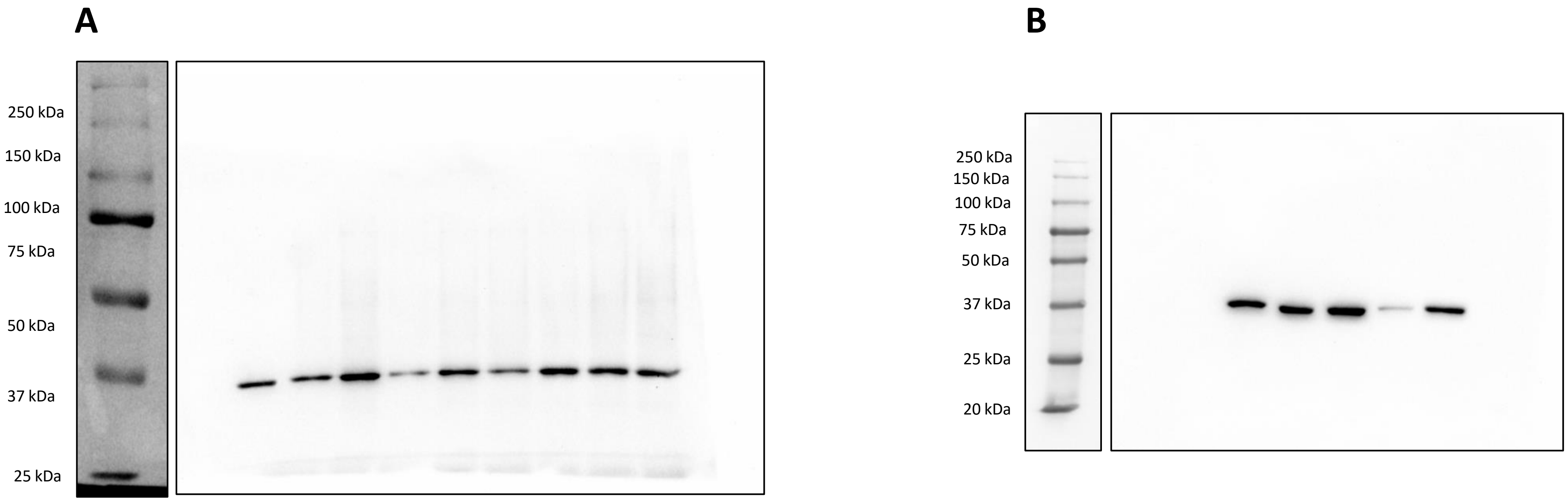

Supplementary Figure 3: Full-length actin blots displayed in Figure 1A and 1B.

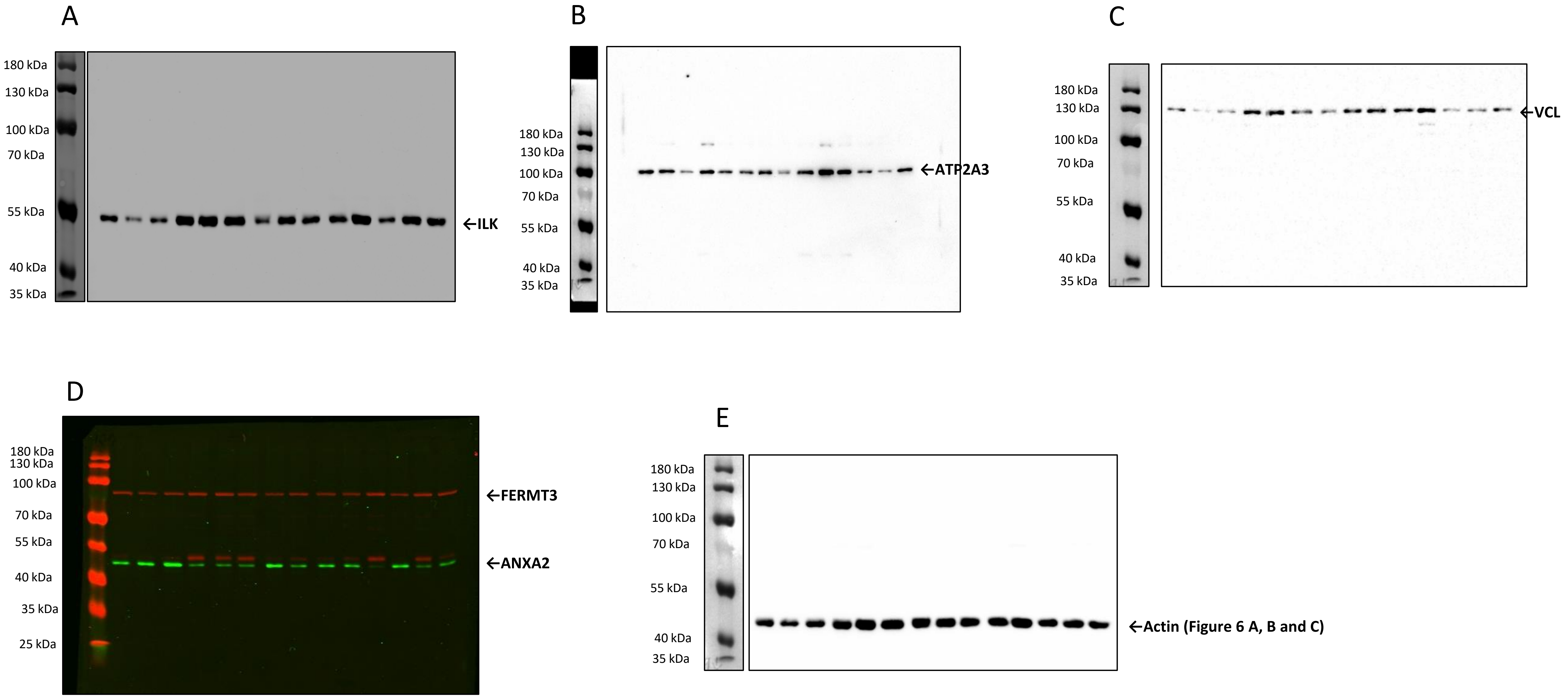

Supplementary Figure 4: Full-length blots displayed in Figure 6. **A:** ILK, **B:** ATP2A3, **C:** VCL, **D:** FERMT3 (red channel), ANXA2 (green channel) and **E:** actin (used as loading control for blots displayed in figure 6 A, B and C. The full-length actin blot used as loading control for Figure 6 D and E is unavailable.
